# First complete female mitochondrial genome in four bivalve species genus *Donax* and their phylogenetic relationships within the Veneroida order

**DOI:** 10.1371/journal.pone.0184464

**Published:** 2017-09-08

**Authors:** Jenyfer Fernández-Pérez, Ana Nantón, Francisco J. Ruiz-Ruano, Juan Pedro M. Camacho, Josefina Méndez

**Affiliations:** 1 Grupo Xenomar, Departamento de Bioloxía, Facultade de Ciencias and CICA (Centro de Investigacións Científicas Avanzadas), Universidade da Coruña, Campus de A Zapateira, A Coruña, Spain; 2 Departamento de Genética, Facultad de Ciencias, Universidad de Granada, Granada, Spain; Tel Aviv University, ISRAEL

## Abstract

**Background:**

Four species of the genus *Donax* (*D*. *semistriatus*, *D*. *trunculus*, *D*. *variegatus* and *D*. *vittatus*) are common on Iberian Peninsula coasts. Nevertheless, despite their economic importance and overexploitation, scarce genetic resources are available. In this work, we newly determined the complete mitochondrial genomes of these four representatives of the family Donacidae, with the aim of contributing to unveil phylogenetic relationships within the Veneroida order, and of developing genetic markers being useful in wedge clam identification and authentication, and aquaculture stock management.

**Principal findings:**

The complete female mitochondrial genomes of the four species vary in size from 17,044 to 17,365 bp, and encode 13 protein-coding genes (including the *atp8* gene), 2 rRNAs and 22 tRNAs, all located on the same strand. A long non-coding region was identified in each of the four *Donax* species between *cob* and *cox2* genes, presumably corresponding to the Control Region. The Bayesian and Maximum Likelihood phylogenetic analysis of the Veneroida order indicate that all four species of *Donax* form a single clade as a sister group of other bivalves within the Tellinoidea superfamily. However, although Tellinoidea is actually monophyletic, none of its families are monophyletic.

**Conclusions:**

Sequencing of complete mitochondrial genomes provides highly valuable information to establish the phylogenetic relationships within the Veneroida order. Furthermore, we provide here significant genetic resources for further research and conservation of this commercially important fishing resource.

## Introduction

Bivalve molluscs of the genus *Donax* (Donacidae family) are an important constituent of the macrofauna of sandy beaches in temperate, tropical and subtropical zones, being the dominant organisms in this type of environment [1]. In the littoral of Iberian Peninsula, the five European species of *Donax* live sympatrically in the same beaches [2, 3]: *D*. *trunculus* (Linnaeus, 1758) (Atlantic and Mediterranean), *D*. *vittatus* (Da Costa, 1778) (Atlantic), *D*. *variegatus* (Gmelin, 1791) (Atlantic and Mediterranean), *D*. *semistriatus* (Poli, 1775) (Atlantic and Mediterranean) and *D*. *venustus* (Poli, 1775) (Atlantic and Mediterranean) [4, 5, 6, 7]. Nevertheless, *D*. *venustus* is practically non-existent in the Iberian Peninsula as a single individual has been found between the years 2000 and 2006 along the south coast of Portugal [3].

Few species of the genus *Donax* are commercially exploited, but some are consumed locally or used as fishing bait. *D*. *trunculus* is exploited in many countries bordering the Mediterranean Sea and Atlantic Ocean, including Portugal [8, 9], Italy [10], France [11], and Spain [12, 13]. Only in Iberian Peninsula, the recorded captures since 1999 to 2014 equal 10,156 tons, with a maximum production of 1,042 tons in 2005 followed by an incessant decline reaching only 250 tons in 2014 [14]. Although this data only reflects production since fishermen were obliged to declare their captures [8], the species has been subjected to intense exploitation over the last decades and, currently, some *D*. *trunculus* populations seem to be at high long-term risk of extinction [15]. Furthermore, this species constitutes an important shellfish resource due to its high economical value. For instance, in Galicia (northwest of Spain), *D*. *trunculus* is a species with a high contribution rate, being the bivalve with greater commercial value (38.52 €/kg in the year 2016) [16] in markets during last years. Due to the similarity in size, shape and colour of the *Donax* clams in different species, captures of *D*. *trunculus* in natural beds may contain other species of the genus with lesser economical value and may be marketed together. However, despite their overexploitation and economic importance, relatively few genetic resources are available for this species [15, 17] and the whole genus [18, 19].

In order to preserve this important fishing resource, genetic tools should be employed. Molecular genetics has proven highly informative to determine the level of genetic variability, which is an essential feature to consider when defining conservation priorities, as well as to better understand the (recent) evolutionary history of species groups. Within the molecular resources, mitochondrial (mt) genome stands out to be considered a useful tool for population genetic and phylogenetic studies, not only because complete mt genomes are often more informative than single genes, but also because they reveal some genome-level details, such as the rearrangement of genes, which are valuable information for studies of evolutionary relationships among species [20, 21, 22, 23]. Moreover, mitochondrial DNA (mtDNA) is particularly important in helping to differentiate species that are morphologically similar, contributing to the identification and authentication of commercial food species to detect and avoid fraud, to protect consumer rights and to achieve other quality objectives, such as certificate of origin.

Most metazoan mitochondrial genomes are typically closed circular molecules of ~16 kb, enconding 37 genes: 13 protein-coding genes (PCGs), 22 transfer RNA (tRNA) genes and two ribosomal RNA (rRNA) genes [24]. In addition, at least one extensive non-coding sequence is present which contain elements that control the initiation of replication and transcription [25]. Mitochondrial genome has several valuable features that make it exclusive, including its small size, high evolutionary rates, limited recombination, relatively conserved gene content and organization, and maternal inheritance [22, 26]. Though, an extreme exception to the paradigm of strict maternal inheritance of animal mtDNA (SMI) is found in some bivalve lineages, which possess an unusual system known as doubly uniparental inhertince (DUI) ([27, 28, 29] for reviews). Species showing DUI display two different kinds of mitochondrial genomes, i.e. male (M) and female (F) mitogenomes. While females have only the F genome, males are heteroplasmic and possess F and M genomes, which the F type predominating in somatic tissues and the M one in gonads [30, 31]. To date, the vast majority of species with DUI which have been reported belong to the orders Mytiloida, Nuculanoida, Unionoida and Veneroida [32], including the wedge clam *D*. *trunculus* [33].

In this study, we determine, for the first time, the complete female mitochondrial (mt) genome sequences in four species of *Donax* from the Iberian Peninsula, and compare them with those of other marine bivalves. In addition, the four newly sequenced mitogenomes, together with the veneroids mt genomes available in GenBank, were used to construct the phylogenetic relationships in the Veneroida order. This work should be of importance not only for better understanding the phylogenetic relationships within the Veneroida order, but also for the development of genetic markers useful in wedge clams aquaculture and restoration effects, as well as for the identification and authentication of commercial species.

## Materials and methods

### Ethics statement

All clams handling was conducted in accordance with the guidelines and regulations established by the University of A Coruña and the local governments. Field sampling did not require specific permissions but was in accordance with general governmental regulations. No endangered or protected species were involved.

### Samples collection and DNA extraction

Given that DUI has been described in *D*. *trunculus* [33] and we have found evidence for it in *D*. *vittatus* and *D*. *semistriatus* [34], and since the goal of our work was on female mtDNA, we used somatic cells of female specimens as the only source for mtDNA sequencing. Therefore, each of the four *Donax* complete mt genomes sequenced here was obtained from a single female specimen in each species, sampled at natural beds. The *D*. *trunculus* sample was collected at Corrubedo (A Coruña, northwestern Spain) while the *D*. *semistriatus*, *D*. *variegatus* and *D*. *vittatus* samples came from the Portuguese coast (Table 1). Gender determination was performed on each individual by microscopic examination of gametogenic tissue from the visceral mass, and was based on the presence of eggs or sperm. Specimens were taxonomically identified using Pereira *et al*. 2012 [18] and Nantón *et al*. 2015 [19] molecular protocols developed in our laboratory. Voucher specimens and their shells were deposited at the malacology collections of the Museo Nacional de Ciencias Naturales (MNCN), Madrid (Spain) (Table 1).

**Table 1:** Sampling details.

Species	Sampling site	Country	Latitude	Longitude	Voucher no.
*D*. *semistriatus*	Monte Gordo	Portugal	37.167	-7.503	15.07/13263
*D*. *trunculus*	Corrubedo	Spain	42.566	-9.039	15.07/13264
*D*. *variegatus*	Monte Gordo	Portugal	37.100	-7.633	15.07/13265
*D*. *vittatus*	Mira-Vagueira	Portugal	40.614	-8.769	15.07/13266

Total genomic DNA was extracted from about 40 mg of ethanol-preserved foot muscle tissue of female specimens using DNAeasy Blood and Tissue Kit (Qiagen, Germany) following manufacturer´s instructions with only a minor modification, namely EB (10mM Tris-Cl, pH 8.5) rather than AE (10mM Tris-Cl, 0.5 mM EDTA, pH 9.0) buffer was used to avoid possible interference of EDTA with Nextera enzyme.

### Molecular procedures and sequencing

The purified genomic DNA was assessed by spectrophotometry (NanoDrop ND-1000, Technologies, Inc.), fluorometry (Qubit HS, Invitrogen, USA) and 1% agarose gel electrophoresis. After quality controls, four libraries (one per species) were prepared using the NEBNext® Ultra™ DNA Library Prep Kit for Illumina® and sequenced in the Illumina HiSeq 4000 platform yielding about 20 Gb data for *D*. *vittatus* and 10 Gb for each of the three other species, subdivided into 2x150 nt paired-end reads.

### Mitogenome assembly and annotation

The mt genomes were reconstructed using 2x1,000,000 reads per species with the MITObim assembler [35]. We performed a first assembly with the -quick option, which resulted in a partial mt genome sequence of about 10,000 bp. In order to get the complete sequence, we extracted the sequence of the COI gene from the previous assembly to be used as starting sequence in MITObim with the -seed option. This yielded sequence of about 17,000 bp whose quality and completeness were assessed on the basis of their average coverage along their whole length, by mapping, in each species, the same 2x1,000,000 reads used in the assembly against the inferred mitogenome sequence. For this purpose, we used the SSAHA2 software [36] with a minimum score of 100. Then we extracted coverage information from these mapping using pysamstats (available at: http://github.com/alimanfoo/pysamstats).

The mt genomes were annotated using the MITOS Web Server [37] applying the invertebrate mitochondrial genetic code and followed by manual validation of the coding regions using the NCBI ORF Finder (https://www.ncbi.nlm.nih.gov/orffinder/). Based on ORF Finder result, the sqn files generated from MITOS were edited and submitted to NCBI. The annotations of PCGs were refined, while the annotations of tRNA genes were kept unchanged. tRNA genes were detected using MITOS, tRNAScan-SE v.2.0 [38] and ARWEN v.1.2 [39]; and secondary structures of tRNAs were inferred using MITOS in default search mode. Mitogenome maps were drawn using GenomeVx online tool [40] followed by manual modification. Repeat sequence patterns in the longest non-coding region (NCR) were checked using the web-based software server Tandem Repeats Finder (http://tandem.bu.edu/trf/trf.html) [41].

### Phylogenetic analyses

To investigate the phylogenetic relationships between species of the Veneroida order, we used the 33 mitogenomes currently available in GenBank (last accessed 17 January 2017), in addition to the four newly determined in this work. *Lucinella divaricata* and *Loripes lacteus*, belonging to the order Lucinoida, were used as outgroups (Table 2). Owing to the fact that a lack of the *Atpase subunit 8* (*atp8*) gene has been reported in some bivalve species, we investigated the possibility that its presence might have gone unnoticed in these species by actively searching for *atp8* sequence in an annotation with MITOS and aligning with other mitogenomes using Geneious Pro v.4.8.5 [42]. We found the *atp8* gene in eight species where previous analyses had concluded the absence of this gene. The alignment of the amino acid sequences for each of the 13 mitochondrial PCGs was performed with the MUSCLE plug-in in Geneious Pro v.4.8.5 [42] with default parameters. We removed poorly aligned regions with Gblocks v.0.91b [43], with options allowing gaps for all positions and 85% of the number of sequences for flanking positions. The 13 separate amino acid sequence alignments were then concatenated into a single large dataset consisting of 2617 sites (S1 File).

**Table 2:** List of the species whose mitogenome sequences were used in the phylogenetic analysis.

Species	Classification	GB Accession no.	Reference
*Donax semistriatus*	Veneroida; Tellinoidea; Donacidae	KY780363	This study
*Donax trunculus*	Veneroida; Tellinoidea; Donacidae	KY780364	This study
*Donax variegatus*	Veneroida; Tellinoidea; Donacidae	KY780365	This study
*Donax vittatus*	Veneroida; Tellinoidea; Donacidae	KY780366	This study
*Macoma balthica*	Veneroida; Tellinoidea; Tellinidae	KM373200	[50]
*Moerella iridescens*	Veneroida; Tellinoidea; Tellinidae	JN398362	[51]
*Nuttallia olivacea*	Veneroida; Tellinoidea; Psammobiidae	JN398364	[51]
*Semele scabra*	Veneroida; Tellinoidea; Semelidae	JN398365	[51]
*Solecurtus divaricatus*	Veneroida; Tellinoidea; Solecurtidae	JN398367	[51]
*Soletellina diphos*	Veneroida; Tellinoidea; Psammobiidae	JN398363	[51]
*Sinonovacula constricta*	Veneroida; Solenoidea; Pharidae	JN398366	[51]
*Solen grandis*	Veneroida; Solenoidea; Solenidae	HQ703012	[56]
*Solen strictus*	Veneroida; Solenoidea; Solenidae	JN786377	[57]
*Cyclina sinensis *	Veneroida; Veneroidea; Veneridae	KU097333	[75]
*Meretrix lamarckii*	Veneroida; Veneroidea; Veneridae	GU071281	[76]
*Meretrix lusoria*	Veneroida; Veneroidea; Veneridae	GQ903339	[62]
*Meretrix lyrata*	Veneroida; Veneroidea; Veneridae	KC832317	[77]
*Meretrix meretrix*	Veneroida; Veneroidea; Veneridae	GQ463598	[78]
*Meretrix petechialis*	Veneroida; Veneroidea; Veneridae	EU145977	[79]
*Paphia amabilis*	Veneroida; Veneroidea; Veneridae	JF969276	[49]
*Paphia euglypta*	Veneroida; Veneroidea; Veneridae	GU269271	[80]
*Paphia textile*	Veneroida; Veneroidea; Veneridae	JF969277	[49]
*Paphia undulata*	Veneroida; Veneroidea; Veneridae	JF969278	[49]
*Ruditapes philippinarum*	Veneroida; Veneroidea; Veneridae	KT001084	[81]
*Saxidomus purpuratus*	Veneroida; Veneroidea; Veneridae	KP419933	[82]
*Acanthocardia tuberculata*	Veneroida; Cardioidea; Cardiidae	DQ632743	[59]
*Fulvia mutica*	Veneroida; Cardioidea; Cardiidae	NC_022194	[83]
*Tridacna squamosa*	Veneroida; Cardioidea; Cardiidae	KP205428	[84]
*Corbicula fluminea*	Veneroida; Corbiculoidea; Corbiculidae	KX254564	Tao et al., unpublished
*Geloina coaxans*	Veneroida; Corbiculoidea; Corbiculidae	KP999913	Zhou, unpublished
*Calyptogena magnifica*	Veneroida; Glossoidea; Vesicomyidae	KR862368	[85]
*Arctica islandica*	Veneroida; Arcticoidea; Arcticidae	KF363951	[86]
*Coelomactra antiquata*	Veneroida; Mactroidea; Mactricidae	KC503290	[87]
*Lutraria rhynchaena*	Veneroida; Mactroidea; Mactricidae	NC_023384	[88]
*Mactra chinensis*	Veneroida; Mactroidea; Mactricidae	KJ754823	[89]
*Lucinella divaricata*	Lucinoida; Lucinoidea; Lucinidae	EF043342	Dreyer et al., unpublished
*Loripes lacteus*	Lucinoida; Lucinoidea; Lucinidae	EF043341	Dreyer et al., unpublished

Phylogenetic analyses were performed under Maximun Likelihood (ML) using RaxML [44] in a web server (http://embnet.vital-it.ch/raxml-bb/) and Bayesian inference (BI) using MrBayes v3.2.6 [45] and PhyloBayes [46]. The best fit models of amino acid evolution were chosen by ProtTest v.3.4.2 [47], with default settings, based on Akaike Information Criterion (AIC). The optimal chosen methods were: LG + I + G + F for *cox1*, *cox3* and *nad5* genes; LG + G + F for *cox2*, *nad6* and *atp8*; MtArt + I + G + F for *cob*, *atp6*, *nad2* and *nad4*; MtArt + I + G + F for *nad1*, *nad3* and *nad4l*. However, as the MtArt evolutionary model is not available in MrBayes, the LG model (the second best-fit model according to ProtTest) was used in Bayesian analysis, being therefore: LG + I + G + F for *cox1*, *cox3*, *cob*, *nad1*, *nad2*, *nad3*, *nad4* and *nad5* genes; LG + G + F for *cox2*, *atp6*, *nad6* and *atp8*; and LG + G for *nad4l*. The ML analyses consisted of 1000 bootstrap iterations using the CAT model for each partition. BI analysis consisted of two independent Markov chain Monte Carlo (MCMC) runs, each comprising four linked chains (one cold and three heated; as default settings). They were performed for 1,000,000 generations, sampling every 100 generations to allow adequate time for convergence. The convergence of the two runs was assessed by stopping the analysis when the average standard deviation was below 0.01 (stoprule = yes and stopval = 0.01 in the mcmc command). 1,000,000 generations were enough to reach adequate average standard deviation (<0.01). By default, the first 25% trees were discarded as burn-in. BI analyses were also conducted at the amino-acid level using the CAT + GTR model in PhyloBayes [46]. Two independent MCMC analyses were run in parallel for 4,000 generations. The first 1,000 samples were discarded as burn-in. From the remaining samples, we sampled a tree every 10 cycles to compute a consensus tree. The convergence between the two chains were considered acceptable when the maxdiff parameter was below 0.3 (maxdiff = 0.218586) and the minimum effective size (MES) was >50 (MES = 64).

## Results and discussion

### Sequencing and mitogenome assembly

A total of about 92,000,000 paired reads (2x150 nt) were obtained for *D*. *semistriatus*, about 85,000,000 for *D*. *trunculus*, about 82,000,000 for *D*. *variegatus* and about 185,000,000 for *D*. *vittatus*. We selected 2x1,000,000 reads that were used to assemble the mitogenome in each species, yielding average coverages of 45x in *D*. *semistriatus*, 31x in *D*. *trunculus*, 37x in *D*. *variegatus*, and 58x in *D*. *vittatus*. Coverage profiles were uniform along the mt genomes (see S1 Fig).

### Genome composition

**Fig 1:**
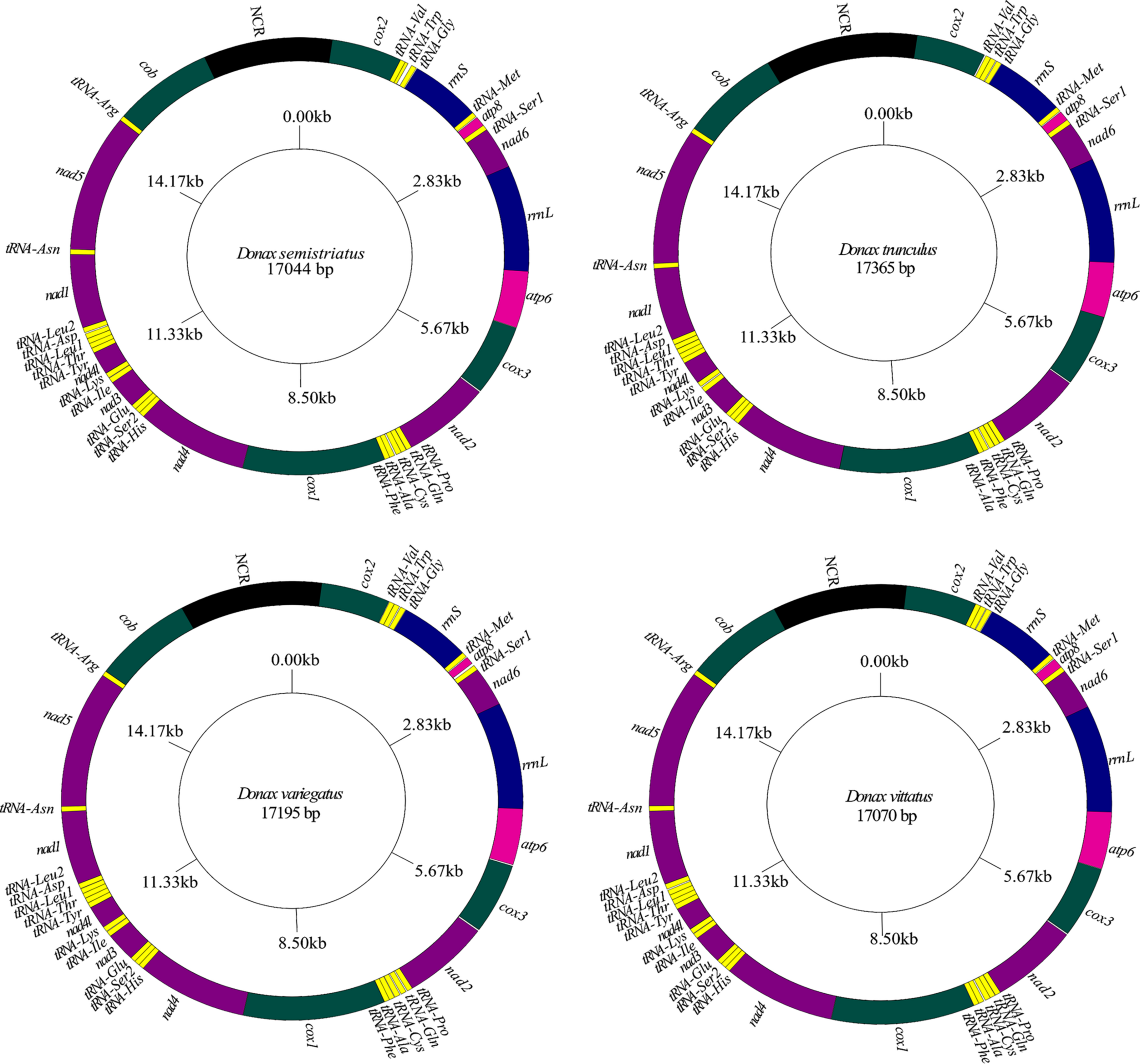
Maps of the mitochondrial genomes of *Donax* species. Genome lengths are shown in the middle of each map, genes are all on “+” strand and NCR indicates the longest non-coding region.

The mitogenomes of the four *Donax* species sequenced in this study were circular molecules, as revealed by the MITObim assembly. They are composed of 37 genes: 13 PCGs (including the *atp8* gene), two ribosomal RNA genes and 22 transfer RNA genes (Fig 1). Their main structural features are summarized in Table 3. The complete mt genomes of *D*. *semistriatus*, *D*. *trunculus*, *D*. *variegatus* and *D*. *vittatus* vary in size from 17,044 bp (*D*. *semistriatus*) to 17,365 bp (*D*. *trunculus*). Length differences are mostly due to the size variation of the non-coding region. The A+T content of the four mitogenomes ranges from 58.9% (*D*. *trunculus*) to 63.5% (*D*. *vittatus*). Although gene organization is known to vary extensively, even among species from the same genus [22, 48, 49], all four complete *Donax* mt genomes showed the same gene order and they are located on the “+” strand, likewise in *Macoma balthica*, other member of the Tellinoidea superfamily for which the whole mt genome is available [50]. The only difference was noted in the location of the longest NCR which, in *M*. *balthica*, is situated between *rrnS* and *tRNA-Met*, whereas in *Donax* clams it is located between *cob* and *cox2* genes (Fig 1). Therefore, in consistency with the highly rearranged gene order in bivalves, the longest NCR is not conserved at the same position among bivalve mt genomes [51, 52].

**Table 3:** Main structural features of the four sequenced mt genomes in this study.

	*Donax semistriatus*	*Donax trunculus*	*Donax variegatus*	*Donax vittatus*
**Total length**	17044	17365	17195	17070
**A+T%**	61.9	58.9	60.4	63.5
*cox2*	846 (ATG/TAA)	846 (ATG/TAA)	831 (ATG/TAG)	846 (ATG/TAA)
*tRNA-Val*	62	64	64	64
*tRNA-Trp*	69	68	69	69
*tRNA-Gly*	64	65	66	66
*rrnS*	863	860	859	865
*tRNA-Met*	65	65	65	65
*atp8*	126 (ATG/TAG)	126 (ATG/TAG)	126 (ATG/TAA)	126 (ATG/TAG)
*tRNA-Ser1*	68	69	69	68
*nad6*	576 (ATG/TAG)	573 (ATG/TAA)	540 (ATG/TAA)	576 (ATG/TAG)
*rrnL*	1373	1367	1383	1386
*atp6*	714 (ATG/TAA)	714 (ATG/TAA)	711 (ATG/TAG)	714 (ATG/TAG)
*cox3*	891 (ATG/TAG)	915 (ATA/TAA)	891 (ATG/TAG)	891 (ATG/TAG)
*nad2*	1062 (ATG/TAA)	1062 (TTG/TAG)	1062 (ATG/TAA)	1062 (ATG/TAA)
*tRNA-Pro*	67	68	67	67
*tRNA-Gln*	65	66	67	65
*tRNA-Cys*	66	66	68	66
*tRNA-Ala*	64	65	66	65
*tRNA-Phe*	63	64	64	63
*cox1*	1710 (ATG/TAA)	1710 (ATG/TAA)	1710 (ATG/TAA)	1710 (ATG/TAA)
*nad4*	1347 (TTG/TAA)	1356 (TTG/TAG)	1332 (TTG/TAA)	1347 (TTG/TAA)
*tRNA-His*	66	66	66	64
*tRNA-Ser2*	66	65	66	65
*tRNA-Glu*	63	64	63	63
*nad3*	363 (ATG/TAA)	363 (ATG/TAA)	363 (ATG/TAA)	363 (ATG/TAA)
*tRNA-Ile*	69	69	69	69
*tRNA-Lys*	65	63	64	64
*nad4l*	288 (TTG/TAG)	288 (TTG/TAG)	288 (ATG/TAA)	288 (TTG/TAG)
*tRNA-Tyr*	64	64	66	65
*tRNA-Thr*	63	65	66	64
*tRNA-Leu1*	65	66	65	65
*tRNA-Asp*	63	62	64	63
*tRNA-Leu2*	65	66	65	66
*nad1*	924 (ATG/TAG)	924 (ATG/TAA)	924 (ATG/TAG)	924 (ATG/TAG)
*tRNA-Asn*	65	64	66	65
*nad5*	1734 (ATG/TAA)	1734 (GTG/TAG)	1734 (ATG/TAA)	1734 (ATG/TAA)
*tRNA-Arg*	63	63	63	63
*cob*	1215 (ATG/TAA)	1218 (ATA/TAA)	1206 (ATG/TAA)	1215 (ATG/TAA)

For each mt genome, total length (in bp), the percent of overall A+T content, and size (bp) of the protein coding genes (start and stop codons in brackets), tRNAs, *rrnL* and *rrnS* are given.

### Protein coding genes

The typical 13 PCGs were identified in the four new mitogenomes analyzed here, including the *atp8* gene, which had been reported as missing in several bivalve species [51, 53, 54, 55, 56, 57, 58], but subsequent analysis found its presence in several of them [48, 50, 52, 59, 60, 61, 62]. It was suggested that the short and variable length of this protein, along with its high variation in amino acid composition, might hinder the finding of this gene due to annotation difficulties [22]. However, using the same bioinformatic approach employed in *Donax* species, we found the *atp8* gene in publicly available mitogenome sequences of most Veneroida order species available in the databases (Table 4). Moreover, we found other discrepancies with GenBank annotations. The *tRNA-Lys* annotation for *Mactra chinensis* (KJ754823) was modified (from 9945–10028 to 13611–13677) and in the following cases, the previous *rrnS* annotations were also edited: *rrnS* for *M*. *meretrix* (GQ463598) and *M*. *petechialis* (EU145977) were edited from 7093–8673 to 7089–8569; for *C*. *antiquata* (KC503290) from 7898–9197 to 7898–9096; and for *L*. *rhynchaena* (NC_023384) from 6870–8244 to 6870–8161.

**Table 4:** Presence of the *atp8* gene in the mitogenomes of the Veneroida order.

Species	*atp8*	Size	Position	Start/Stop codons	Reference
*Donax semistriatus*	Yes	126	2396–2521	ATG/TAG	This study
*Donax trunculus*	Yes	126	2419–2544	ATG/TAG	This study
*Donax variegatus*	Yes	126	2352–2477	ATG/TAA	This study
*Donax vittatus*	Yes	126	2310–2435	ATG/TAG	This study
*Macoma balthica*	Yes	129	75–203	ATT/TAA	[50]
*Moerella iridescens*	Yes	132	11625–11756	ATA/TAG	[52]
*Nuttallia olivacea*	Yes	132	12930–13061	ATA/TAG	[52]
*Semele scabra*	Yes	129	11969–12100	ATT/TAA	[52]
*Solecurtus divaricatus*	Yes	135	11321–11455	GTG/TAG	[52]
*Soletellina diphos*	Yes	135	11214–11342	GTG/TAG	[52]
*Sinonovacula constricta*	Yes	114	14288–14401	ATG/TAA	This study
*Solen grandis*	Yes	114	13703–13816	GTG/TAG	This study
*Solen strictus*	Yes	114	13473–13586	ATG/TAG	This study
*Cyclina sinensis *	Yes	117	8568–8684	ATG/TAA	[75]
*Meretrix lamarckii*	Yes	120	8835–8954	ATG/TAA	[76]
*Meretrix lusoria*	Yes	120	8642–8761	ATG/TAG	[62]
*Meretrix lyrata*	Yes	120	8753–8872	ATG/TAG	[77]
*Meretrix meretrix*	Yes	141	8532–8672	ATA/TAG	[52]
*Meretrix petechialis*	Yes	141	8532–8672	ATA/TAG	[52]
*Paphia amabilis*	Yes	114	14035–14148	ATG/TAG	[49]
*Paphia euglypta*	Yes	117	12994–13110	ATA/TAA	[52]
*Paphia textile*	Yes	114	13019–13132	ATG/TAA	[49]
*Paphia undulata*	Yes	114	12642–12755	ATG/TAA	[49]
*Ruditapes philippinarum*	Yes	120	5968–6087	ATT/TAG	[52]
*Saxidomus purpuratus*	Yes	117	9557–9673	ATG/TAA	This study
*Acanthocardia tuberculata*	Yes	103	12546–12648	GTG/CCT	[52]
*Fulvia mutica*	Yes	114	11341–11454	TTG/TAA	[83]
*Tridacna squamosa*	Yes	117	8525–8641	ATG/TAG	This study
*Corbicula fluminea*	Yes	114	5480–5593	ATG/TAA	Tao et al., unpublished
*Geloina coaxans*	Yes	114	12249–12362	TTG/TAG	Zhou, unpublished
*Calyptogena magnifica*	Yes	114	5440–5553	ATG/TAA	[85]
*Arctica islandica*	Yes	151	10343–10493	TTG/AGT	[52]
*Coelomactra antiquata*	Yes	114	9097–9210	ATG/TAA	This study
*Lutraria rhynchaena*	Yes	118	8162–8275	ATG/TAA	This study
*Mactra chinensis*	Yes	114	10000–10113	ATG/TAG	This study
*Lucinella divaricata*	Yes	114	15861–15974	ATT/TAA	Dreyer et al., unpublished
*Loripes lacteus*	Yes	118	14442–14589	ATT/ACT	Dreyer et al., unpublished

For each *atp8* sequence, size (bp), position (from-to), and start and stop codons.

The location of the *atp8* gene within the mitogenome is the same in the eight species of the Tellinoidea superfamily (all four *Donax* species, *M*. *balthica*, *M*. *iridescens*, *S*. *divaricatus* and *S*. *diphos*), i.e. between *tRNA-Met* and *tRNA-Ser1*. In *Donax* species, this short gene encoded a 42 amino acids protein starting with methionine (ATG, in the four species) and ending with a stop codon (TAG in *D*. *semistriatus*, *D*. *trunculus* and *D*. *vittatus*; or TAA, in *D*. *variegatus*) (Table 4), so that ATP8 proteins show 83.7% amino acid identity among species. Finally, it has been suggested that the *atp6* and *atp8* genes are adjacent in most animal mitochondrial genomes, often with overlapping reading frames [63]. However, in *Donax* species *atp6* and *atp8* genes are physically separated by 1,917 (*D*. *trunculus*)– 1,928 bp (*D*. *vittatus*). Likewise, these two genes also fail to be adjacent in the mitogenome of other heterodont bivalves, such as *Hiatella arctica* [59], *M*. *balthica* [50] and *Meretrix lamarckii* [64]. On the contrary, they are adjacent in the Unionidae [65] and Solemydae [66], as well as in basal molluscs like *Chaetoderma nitidilum* (EF211990) and *Katharina tunicata* [67]. This suggests that the association of these genes might be an example of an ancestral state that has later been lost in derived bivalves.

Total length of the 13 PCGs ranged from 11,718 bp (*D*. *variegatus*) to 11,829 bp (*D*. *trunculus*), accounting for 68.1–69.2% of its total mt genome length. The longest PCG is *nad5*, with a size of 1,734 bp (577 aa), whereas *nad2*, *cox1*, *nad4* and *cob* exceed 1,000 bp. However, *nad3* and *nad4l* genes are shorter than 400 bp and *atp8* gene is the shortest PGC with 126 bp (41 aa). These features are similar to those previously reported in *M*. *balthica* [50] and five other species of the Tellinoidea superfamily (*Moerella iridescens*, *Sanguilonaria diphos*, *Sanguinolaria olivacea*, *Semele scabra* and *Solecurtus divaricatus*) [51].

The ATN conventional start codon is used in most PCGs (ATG, N = 41; ATA, N = 2; the last codon being classically found in the invertebrate mitochondrial genetic code, particularly in bivalves [50]). However, like most invertebrate mt genomes, *Donax* mtDNA shows alternative start codons, and some PCGs start with NTG codons (TTG, N = 8; GTG, N = 1). In contrast, the observed stop codons are TAA (N = 32) and TAG (N = 20), and all 13 PCGs of the four mt genomes end in a full termination codon.

### Transfer and ribosomal RNA genes

Standard rRNAs were found in the four mt genomes of *Donax* species analyzed here. The small-subunit ribosomal RNA (*rrnS*) was flanked by *tRNA-Gly* and *tRNA-Met* in all four mt genomes, and its size ranged from 859 bp (*D*. *variegatus*) to 865 bp (*D*. *vittatus*), with A+T content between 63.8 (*D*. *semistriatus*) and 68.5% (*D*. *vittatus*). On the other hand, the large-subunit ribosomal RNA (*rrnL*) was located between *nad6* and *atp6*, just like in *M*. *balthica* [50], *M*. *iridescens*, *S*. *diphos*, *S*. *olivacea*, *S*. *scabra*, *S*. *constricta* and *S*. *divaricatus* [51]. Its size varied from 1,367 bp (*D*. *semistriatus*) to 1,386 bp (*D*. *vittatus*), and its A+T content ranged between 63.5 (*D*. *variegatus*) and 67.2% (*D*. *semistriatus*).

Twenty-two discrete nucleotide sequences (ranging from 62 to 69 bp) were predicted to fold into the typical secondary structures of tRNAs (see S2*–*S5 Figs). The predicted structures of tRNA genes showed cloverleaf shape with four arms in the four species, although some of them exhibited folding differences. Sixteen *tRNAs* showed a small supplemental stem loop (four in *D*. *semistriatus*: t*RNA-Pro*, *tRNA-Phe*, *tRNA-Ile* and *tRNA-Leu2*; two in *D*. *trunculus*: *tRNA-Ile* and *tRNA-Thr*; six in *D*. *variegatus*: t*RNA-Val*, t*RNA-Pro*, *tRNA-Gln*, *tRNA-His*, *tRNA-Ile* and *tRNA-Arg*; and four in *D*. *vittatus*: t*RNA-Pro*, *tRNA-Phe*, *tRNA-Ile* and *tRNA-Leu2*). Seven *tRNAs* showed no terminal TΨC loop (three in *D*. *semistriatus*: t*RNA-His*, *tRNA-Thr* and *tRNA-Arg*; one in *D*. *trunculus*: *tRNA-Asn*; and three in *D*. *vittatus*: t*RNA-His*, *tRNA-Thr* and *tRNA-Asp*). In addition, *tRNA-Ser2* in *D*. *trunculus* showed the dihydrouracil (DHU) stem replaced by a big DHU loop. Finally, the single unpaired nucleotide, which is usually present at the 5´end in other tRNAs, appeared at the 3´end in *tRNA-Tyr*, with the only exception of *D*. *variegatus* where this tRNA lacks this unpaired nucleotide. These features have previously been found in mtDNAs of other bivalve species, such as *M*. *balthica* [50] and *M*. *lamarckii* [64].

### Non-coding regions

As in most bivalves, the four species of the genus *Donax* analyzed here contained a large number of NCRs. The number of intergenic sequences varied from 17 (*D*. *trunculus* and *D*. *vittatus*) to 22 (*D*. *variegatus*), with 1,679 bp (representing 9.9% of the whole mitogenome) in *D*. *semistriatus* to 1,985 bp (11.4% of the mt genome) in *D*. *trunculus* (Table 5). The longest NCR was located between *cob* and *cox2* genes in the four species, with length ranging from 1,549 bp (*D*. *semistriatus*) to 1,863 bp (*D*. *trunculus*). The other NCRs ranged fom 1 to 21 bp. The longest NCR is thought to contain the Control Region (CR) because it presents some peculiar patterns, such as AT-rich or tandem repeats, believed to play a role in initiating and/or regulating mitochondrial transcription and replication [24, 68, 69]. The A+T content of the longest NCR in each mt genome was higher (*D*. *semistriatus*, *D*. *variegatus and D*. *vittatus)* or slightly lower (*D*. *trunculus*) than that of the whole mt genome (Table 5).

**Table 5:** Comparison of non-coding regions (NCRs) within the four mt genomes.

				Longest NCR	
Species	No. of NCR	Total length (bp)	Proportion of the mt genome (%)	Length (bp)	A+T %
*Donax semistriatus*	18	1679	9.9	1549	66.6
*Donax trunculus*	17	1985	11.4	1863	51.8
*Donax variegatus*	22	1869	10.9	1718	62.6
*Donax vittatus*	17	1697	9.9	1580	67.5

Six tandem repeats were also found in the longest NCRs of the four mt genomes, four of which were distinct tandem repeat units. The first motif consisted of 2.7 nearly identical copies of a 122 bp unit located at positions 48–386 from the 5´-end of the longest NCR in *D*. *semistriatus*. The second was 2.1 copies of 126 bp located at positions 17042–17309 in *D*. *trunculus*. In addition, microsatellite-like repeats, (TA)_12_ in *D*. *semistriatus* and (TA)_12_ACACTTGTGA(TA)_10_ in *D*. *trunculus*, were detected near the 5´-end of the longest NCR. The third tandem repeat consisted in 2.1 copies of 137 bp located between positions 57 and 344 in *D*. *variegatus*, and the last one included 2 copies of 122 bp located at positions 47–304 in *D*. *vittatus*. Such long tandem repeats have also been reported in other bivalves of the Veneroida order [51, 55, 59, 62]. The study of tandem repeats in the CR is important for the light it sheds on a variety of processes, including the molecular mechanisms arising them and their possible functional implications [70].

### Phylogenetic analysis in Veneroida

**Fig 2:**
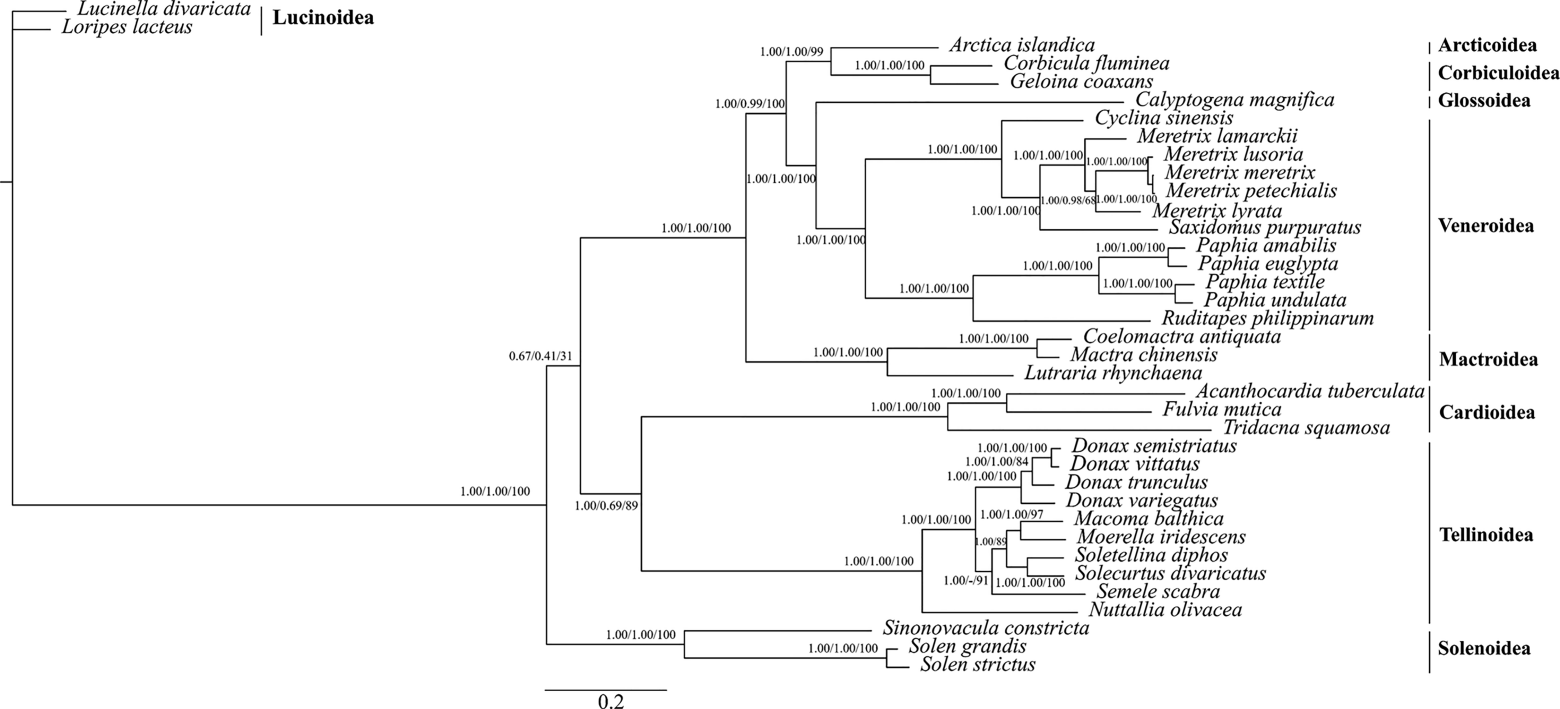
Phylogenetic tree of the Veneroida order based on concatenated amino acids of 13 protein-coding genes. Numbers at the nodes correspond to Bayesian posterior probabilities (left), PhyloBayes posterior probabilities (middle) and ML bootstrap proportions (right). Dash indicates the difference in the position for *S*. *scabra* in the PhyloBayes phylogeny.

To further study the relationships among *Donax* species and its position within the Veneroida order, ML and BI trees based on amino acid sequences of 13 concatenated PCGs belonging to 37 species were performed (Fig 2). Tree topologies were congruent and received high support in most nodes, with the exception of *S*. *scabra*, which showed a less basal position in the PhyloBayes phylogeny ((*M*. *balthica* + *M*. *iridescens)* + *S*. *scabra*) with 0.57 posterior probability as branch support.

We perform here the first phylogeny including the species of the genus *Donax* from the Iberian Peninsula (*D*. *trunculus*, *D*. *semistriatus*, *D*. *variegatus* and *D*.*vittatus*). Our analysis has shown that the four species form a single clade as a sister group to other bivalves of the superfamily Tellinoidea. All ten species of this superfamily belong to five different families and form a strongly supported clade, thus corroborating the monophyly of this superfamily [71, 72]. Nevertheless, our phylogenetic tree indicated, with high support by BI and ML, that *S*. *diphos* (Psammobiidae) shows closer relationship with *S*. *divaricatus* (Solecurtidae), *M*. *balthica* and *M*. *iridescens* (Tellinidae), *S*. *scabra* (Semelidae) and *Donax* species (Donacidae) rather than with *N*. *olivacea* (Psammobiidae), which implies that these two species (*S*. *diphos* and *N*. *olivacea*) do not form monophyletic groups. This result is also reported by Yuan *et al*. 2012 [51] and Ozawa *et al*. 2017 [73], and it is in agreement with the conclusion put forward by Taylor *et al*. 2007 [71] when analysed familial relationships within Tellinoidea, as Semelidae, Donacidae and Tellinidae do not form monophyletic groups. Tellinoidea is actually monophyletic, but none of its families are monophyletic [72], suggesting the need for a more exhaustive study within this commercially important marine bivalve clade.

Gene arrangement within mitogenomes is highly conserved in many taxonomic groups. For instance, most vertebrates share the same gene order [74]. However, in other animal groups, like the class Bivalvia, the mitochondrial genome arrangement is more variable [51, 52]. We compare here the gene arrangements of four newly sequenced mitogenomes to other closed related species belonging to Tellinoidea superfamily. This comparison was previously done by by Yuan *et al*. (2012), without taking into account the *atp8* gene and without including *Donax* species and *M*. *balthica*, and their results supported the conclusion that comparisons of mitochondrial gene order rearrangements are, to some extent, a useful tool for phylogenetic studies. Seven out of the ten Tellinoidea mitogenomes hitherto analyzed (including the four *Donax* species analyzed by us, *M*. *balthica*, *M*. *iridescens* and *S*. *divaricatus*) show completely identical gene order, and *S*. *diphos* only differs in lacking a *tRNA-Phe*. Remarkably, the *atp8* gene shows the same location within the mitogenome of these eight species of the Tellinoidea superfamily, specifically between *tRNA-Met* and *tRNA-*Ser1. This result is consistent with the main phylogenetic conclusions from the 37 mitogenomes analyzed here (see above), and remarks the interest of performing additional full mitogenome sequencing, especially including more veneroid families and subfamilies, with gene order being a useful hallmark helping to clarify phylogenetic relationships within the order.

## Future implications

This is a basic research work where we describe and characterize, for the first time, the female mitochondrial genome in four bivalve molluscs belonging to the genus *Donax*. This has provided new interesting information for the scientific community which can be feasible for application in aquaculture. In fact, the mtDNA sequences contributed here add significantly useful genetic markers for i) helping to differentiate these commercial food species being morphologically similar, ii) detecting and avoiding fraud, iii) protecting consumer rights and achieving other quality objectives, such as certificate of origin, and iv) for using in population genetics studies and aquaculture stock management in *Donax* species. However, this possible applicability requires a broader work, where the different markers will be tested in a higher number of individuals, not only fresh individuals but also processed, packaged or frozen ones, as well as in a high number of females and males given that male genomes are still not available.

## Conclusions

In this study, we determined the complete mt genomes of four bivalve species of the genus *Donax*, which are the first representatives from the family Donacidae being analyzed at this respect. Not only we have increased the number of complete mt genomes sequenced within Veneroida order, but also, we have illustrated the phylogenetic relationships among *Donax* species and their position within this order. Our results demonstrate that the sequencing of complete mitogenomes provides highly valuable information for phylogenetic analysis in bivalves. Furthermore, the mtDNA sequences contributed here add significantly useful genetic markers for use in species identification and authentication, phylogeny, population genetics, and aquaculture stock management in species of *Donax*.

## Supporting information

S1 File The alignment of 37 mitogenomes sequences used for phylogenetic analyses. Sequences include concatenated thirteen mitochondrial protein-coding genes.
(FAS)

S1 Fig Coverage profiles for the four newly sequenced mitochondrial genomes. Blue line represents coverage along the mitochondrial sequences for the four *Donax* species. Red dashed lines represent the average coverage values: 45.46x in *D*. *semistriatus*, 30.94x in *D*. *trunculus*, 37.12x in *D*. *variegatus*, and 58.10x in *D*. *vittatus*.
(TIFF)

S2 Fig Predicted tRNA structures in *D*. *semistriatus*. 22 tRNAs are identified in the mitogenome of *D*. *semistriatus* and their cloverleaf secondary structures are inferred with MITOS annotation pipeline.
(TIF)

S3 Fig Predicted tRNA structures in *D*. *trunculus*. 22 tRNAs are identified in the mitogenome of *D*. *trunculus* and their cloverleaf secondary structures are inferred with MITOS annotation pipeline.
(TIF)

S4 Fig Predicted tRNA structures in *D*. *variegatus*. 22 tRNAs are identified in the mitogenome of *D*. *variegatus* and their cloverleaf secondary structures are inferred with MITOS annotation pipeline.
(TIF)

S5 Fig Predicted tRNA structures in *D*. *vittatus*. 22 tRNAs are identified in the mitogenome of *D*. *vittatus* and their cloverleaf secondary structures are inferred with MITOS annotation pipeline.
(TIF)
