# Supplementary figures and images for: First complete female mitochondrial genome in four bivalve species genus Donax and their phylogenetic relationships within the Veneroida order
Source: PLoS One. 2017 Sep 8;12(9):e0184464. doi: 10.1371/journal.pone.0184464 (PMC5590976; doi:10.1371/journal.pone.0184464)

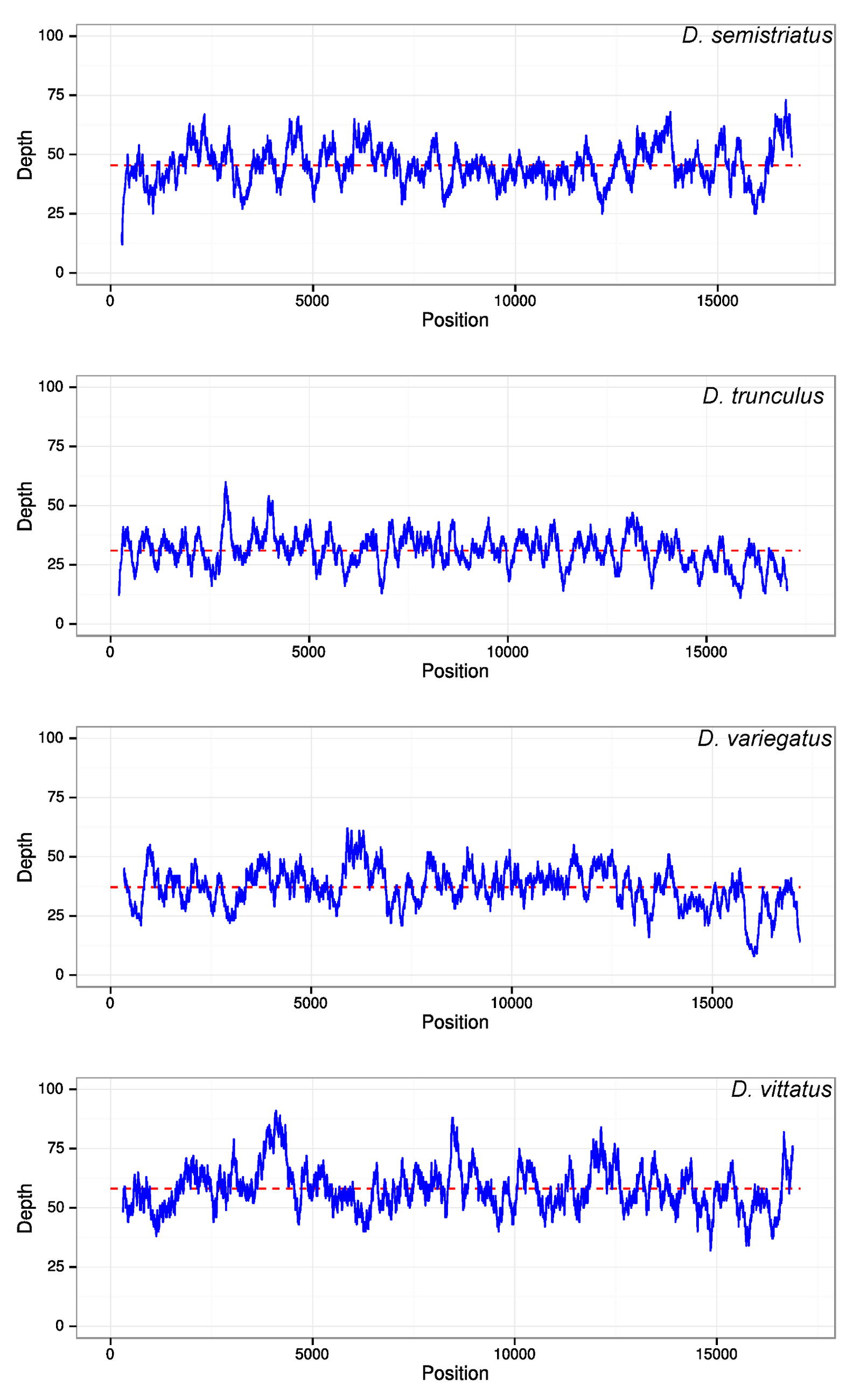

Supplement: S1 Fig — Blue line represents coverage along the mitochondrial sequences for the four Donax species. Red dashed lines represent the average coverage values: 45.46x in D. semistriatus, 30.94x in D. trunculus, 37.12x in D. variegatus, and 58.10x in D. vittatus. (TIFF) [file pone.0184464.s002.tiff]

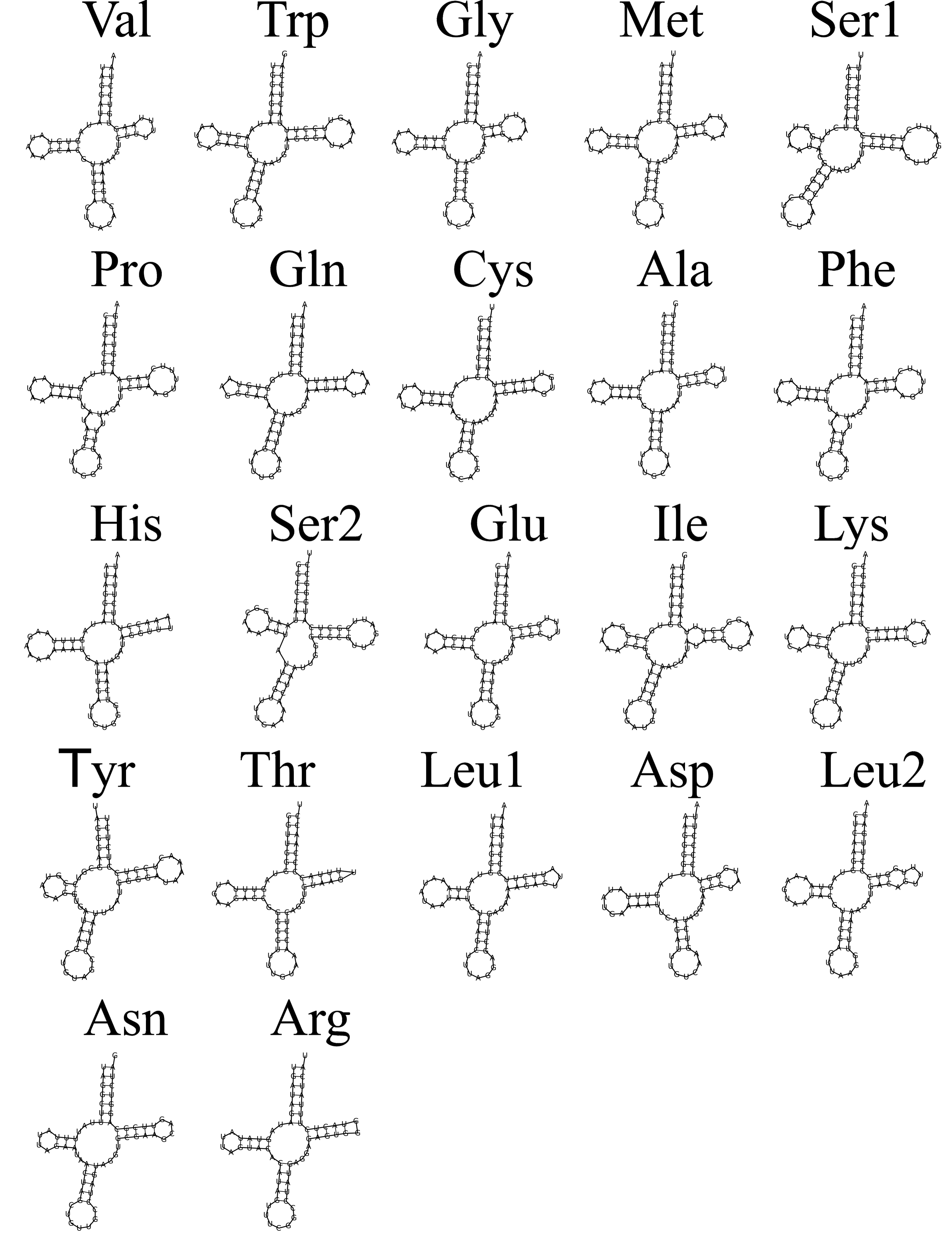

Supplement: S2 Fig — 22 tRNAs are identified in the mitogenome of D. semistriatus and their cloverleaf secondary structures are inferred with MITOS annotation pipeline. (TIF) [file pone.0184464.s003.tif]

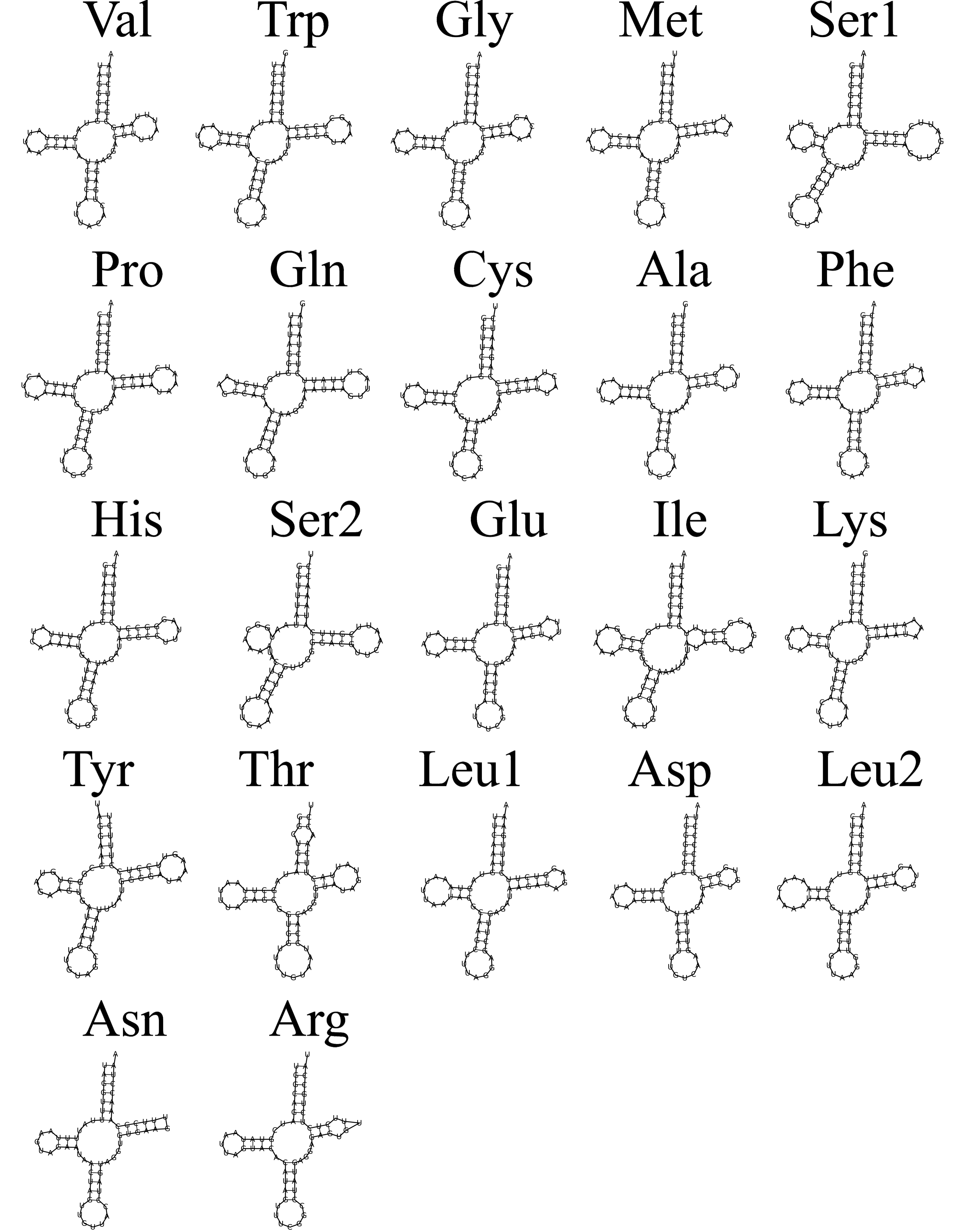

Supplement: S3 Fig — 22 tRNAs are identified in the mitogenome of D. trunculus and their cloverleaf secondary structures are inferred with MITOS annotation pipeline. (TIF) [file pone.0184464.s004.tif]

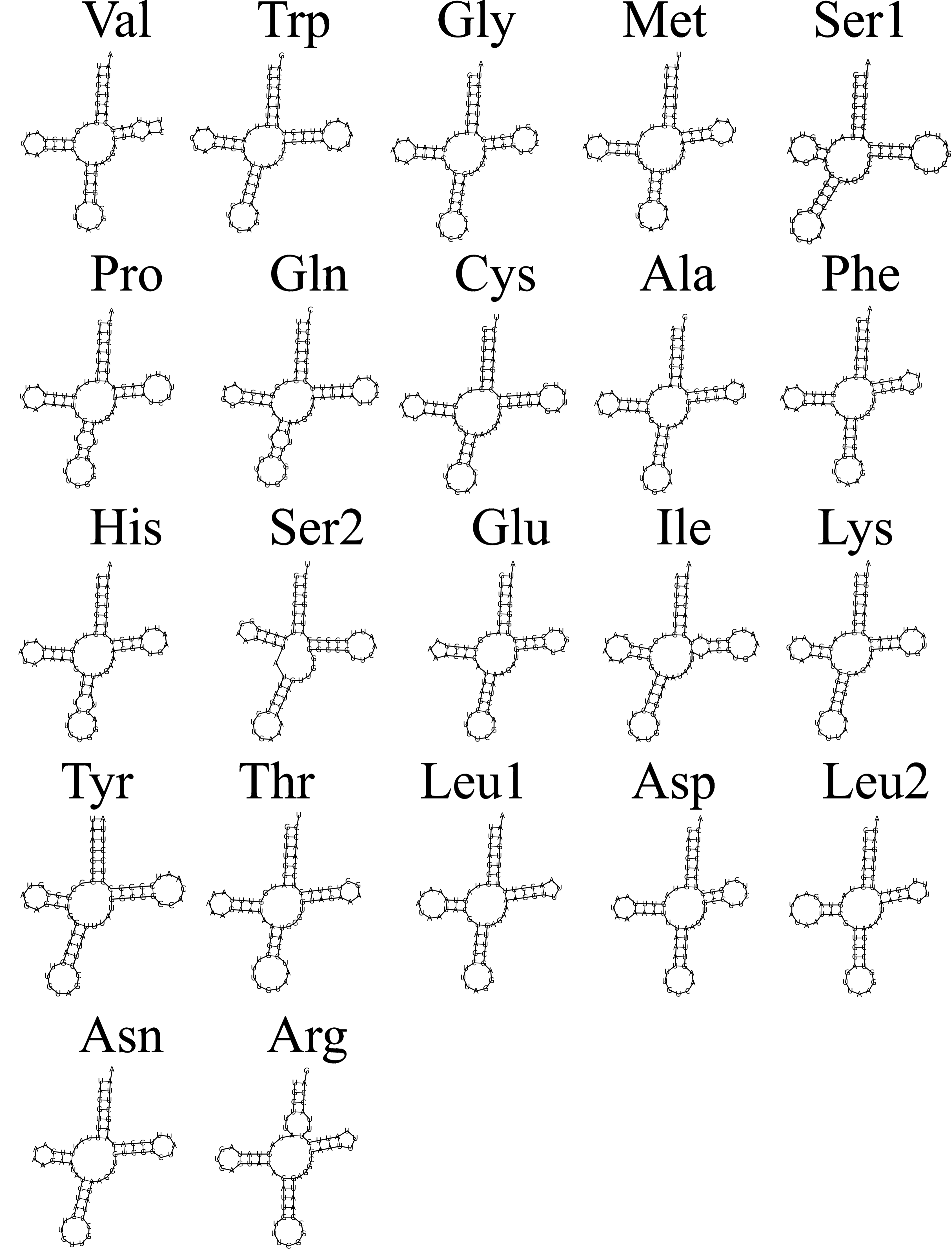

Supplement: S4 Fig — 22 tRNAs are identified in the mitogenome of D. variegatus and their cloverleaf secondary structures are inferred with MITOS annotation pipeline. (TIF) [file pone.0184464.s005.tif]

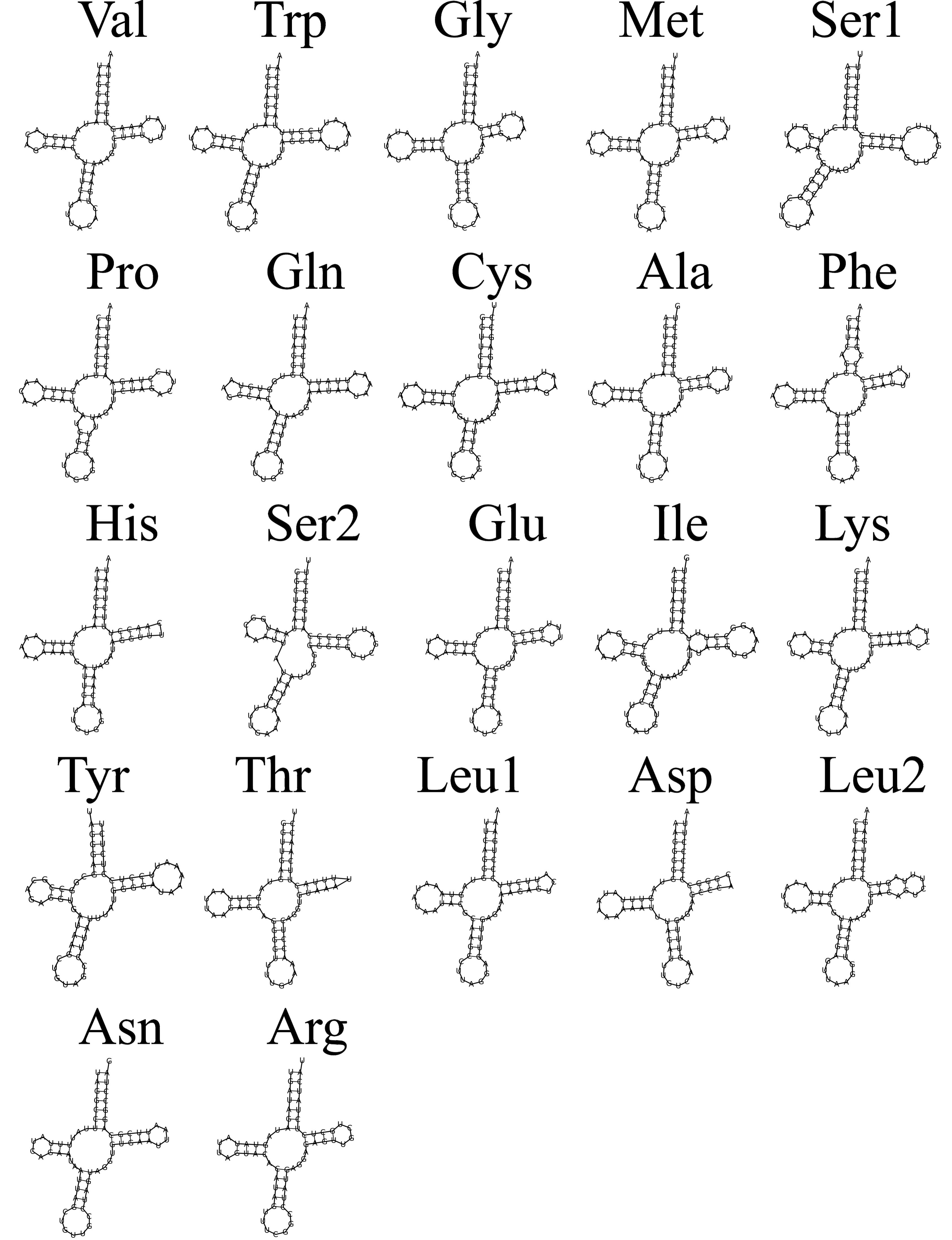

Supplement: S5 Fig — 22 tRNAs are identified in the mitogenome of D. vittatus and their cloverleaf secondary structures are inferred with MITOS annotation pipeline. (TIF) [file pone.0184464.s006.tif]
